# Contrasting Seed Protein Phenotypes Are Associated with Nodulation and Rhizosphere Microbiome Assembly in Soybean

**DOI:** 10.3390/microorganisms14102266

**Published:** 2026-10-05

**Authors:** Jiyuan Guo, Lixue Wang, Song Liu, Hong Xue, Weiwei Zhang, Xuepeng Fu, Quanzhong Dong

**Affiliations:** 1 Department of Life Science and Agroforestry, Qiqihar University, Qiqihar 161006, China; guojiyuan677@gmail.com (J.G.);; 2 School of Life Sciences, College of Heilongjiang Province, Heilongjiang University, Harbin 150080, China; 3 Northeast Institute of Geography and Agroecology, Chinese Academy of Sciences, Harbin 150081, China; 4 Keshan Branch Heilongjiang Academy of Agricultural Sciences, Qiqihar 161601, China

**Keywords:** soybean, seed protein, rhizosphere microbiome, root nodules, microbial community assembly, co-occurrence network

## Abstract

Soybean seed protein content varies with genotype, nitrogen acquisition, and environmental conditions. Associations between belowground microbiota and seed protein content remain insufficiently characterized. We compared three high-protein cultivars (KD91, SN148, and KD80) and three low-protein cultivars (KD122, KD96, and BD40) to examine relationships among seed composition, nodulation, and root-associated microbial communities. High-protein cultivars produced more nodules, while nodule bacterial diversity and community composition showed no significant between-group differences and were dominated by *Bradyrhizobium*. In contrast, rhizosphere bacterial and fungal communities differed between protein groups. *Dyadobacter*, *Pseudoxanthomonas*, and *Chitinophaga* had higher relative abundance in the high-protein rhizosphere. Seed protein content was positively associated with *Dyadobacter* (*R*^2^ = 0.234, *p* = 0.017) and *Pseudoxanthomonas* (*R*^2^ = 0.432, *p* < 0.001), but not with *Chitinophaga*. This study compared distinct cultivars grown in a single batch of peat-based greenhouse substrate; the observed microbial patterns are associated with cultivar group and cannot be attributed to seed protein phenotype alone. *Dyadobacter* and *Pseudoxanthomonas* are candidates for future functional validation.

## 1. Introduction

Soybean (*Glycine max* (L.) Merr.) is one of the world’s most important leguminous crops and a major source of plant-derived protein and edible oil. Seed protein content is therefore a key quality trait and an important target in soybean breeding. However, seed protein accumulation is a complex quantitative trait controlled by genetic background and environmental conditions, and substantial variation occurs among cultivars [1,2,3,4,5]. Because nitrogen is an essential constituent of amino acids and proteins, nitrogen acquisition, assimilation, and allocation to developing seeds are central to the formation of high-protein phenotypes [1,2,5,6]. Although considerable progress has been made in elucidating the genetic basis of soybean seed protein content, the contribution of belowground biological processes, particularly plant-associated microorganisms, to variation in seed protein accumulation remains insufficiently understood.

As a legume with a high nitrogen demand, soybean establishes symbiosis with nitrogen-fixing rhizobia and forms specialized root nodules in which atmospheric N2 is converted into biologically available nitrogen [6,7,8]. Symbiotic nitrogen fixation can supply a substantial proportion of the nitrogen required for soybean growth and seed development, linking nodulation and nitrogen acquisition to seed protein formation. Nodulation and nitrogen-fixation efficiency are influenced by rhizobial strain, environmental conditions, and soybean genotype [7,8,9,10]. Soybean nodules are generally dominated by compatible rhizobia, particularly *Bradyrhizobium*, while lower-abundance nodule-associated bacteria may also influence host performance [11,12,13]. Experimental studies further show that co-inoculation of *Bradyrhizobium* with plant growth-promoting bacteria can enhance nodulation, nitrogen fixation, plant growth, and yield, although responses depend on the host genotype and microbial combination [11,12,14,15]. Nevertheless, whether contrasting seed protein phenotypes are accompanied by differences in nodulation and nodule-associated microbial communities remains unclear.

Beyond nodules, the rhizosphere is a highly dynamic interface in which plant roots interact with diverse soil microorganisms. Plants can selectively shape their rhizosphere microbiota through root-derived resources and signaling compounds, whereas recruited microorganisms can influence nutrient mobilization, nitrogen cycling, phytohormone balance, root development, stress tolerance, and plant health [16,17,18,19,20,21,22,23,24,25,26,27,28,29,30]. Soybean genotype has been shown to contribute to rhizosphere microbiome assembly and cultivar-specific microbial recruitment [16,17]. Importantly, emerging evidence suggests that rhizosphere microbiota may also be linked to crop quality traits. Han et al. demonstrated across a broad soybean panel that oil-related quality traits were associated with rhizosphere microbiome composition and that high-oil cultivars selectively enriched *Massilia* through genotype-dependent root exudation; subsequent inoculation experiments showed that *Massilia* could increase seed oil content [31]. This work provides a compelling precedent that soybean quality traits can be coupled to selective microbial recruitment and raises the possibility that contrasting seed protein phenotypes may likewise be associated with specific rhizosphere microorganisms. However, microbial taxa associated with variation in soybean seed protein content remain poorly characterized.

Microbial community composition alone may not fully explain ecological differences among soybean cultivars. Community assembly analyses can estimate deterministic and stochastic processes, whereas co-occurrence networks describe statistical association patterns and may help prioritize taxa for further study [32,33,34]. In the present study, we compared three high-protein soybean cultivars (KD91, SN148, and KD80) with three low-protein cultivars (KD122, KD96, and BD40). We characterized seed traits, nodulation, nodule-associated bacteria, and rhizosphere bacterial and fungal communities, then compared community assembly and network patterns between the two cultivar groups. We also examined associations between bacterial genus relative abundance and seed protein values. Because each protein group contains three distinct cultivars, protein class cannot be separated from genetic background in this design. Our aim was to identify cultivar-associated microbial patterns and candidate taxa for later validation, not to infer that protein content caused microbiome differences.

## 2. Materials and Methods

### 2.1. Plant Materials and Growth Substrate

Six soybean cultivars were used. The high-protein group comprised KD91, KD80, and SN148, and the low-protein group comprised BD40, KD96, and KD122. Soybean plants were grown in a peat-based growth substrate in a greenhouse at Qiqihar University. Plants were cultivated in a greenhouse maintained at 25–27 °C, using pots with a radius of 15 cm. No additional fertilizer was applied. Seeds were sown at a density of five seeds per pot, and seedlings were thinned to two plants per pot after emergence.

All six soybean cultivars were grown in the same batch of peat-based growth substrate. Four biological replicate groups were established for each cultivar, with each group comprising three pots. For each sample type, material from the three pots within a group was pooled separately and homogenized to form one composite biological sample. Physicochemical measurements of the growth substrate were performed in triplicate. Substrate pH was measured in a substrate-to-water suspension (1:2.5, *w*/*v*). Total nitrogen and hydrolyzable nitrogen were quantified using titration-based procedures, total phosphorus and available phosphorus were determined colorimetrically after extraction, total potassium and available potassium were quantified from extract concentrations, and organic matter was determined by dichromate oxidation followed by ferrous sulfate titration. The physicochemical properties of the experimental growth substrate are summarized in Table 1.

### 2.2. Determination of Seed Total Nitrogen

Seeds were collected at the seed-filling stage. For each biological replicate, seeds from the corresponding three pots were pooled and homogenized to form one composite sample. Four biological replicates were collected for each cultivar. Samples were rapidly frozen in liquid nitrogen, transported to the laboratory, and stored at −80 °C for approximately one week. The samples were allowed to thaw naturally before drying at 70 °C to constant mass (moisture < 5%), after which they were ground and passed through a 40-mesh sieve. For each sample, 0.5 g powder was digested with 5 mL concentrated H_2_SO_4_ and 3 g catalyst mixture (K_2_SO_4_:CuSO_4_:Se = 100:10:1, *w*/*w*/*w*). Digestion was conducted at 240 °C for 30 min and then at 400 °C for 45 min. Total nitrogen was quantified using a Kjeldahl nitrogen analyzer with boric acid/methyl red–bromocresol green receiving solution and 40% NaOH.

### 2.3. Seed Protein, Oil, Soluble Protein, and Amino-Acid Profiling

Seed chemical composition was measured using a DA 7250 near-infrared analyzer (PerkinElmer, Inc., Waltham, MA, USA) with its built-in default calibration settings. NIR seed samples were collected from the greenhouse-grown cultivars during the same seed-filling period as the samples used for total-nitrogen analysis. They were processed using the same freezing, storage, natural thawing, and 70 °C oven-drying procedure described in Section 2.2. For each biological replicate, seeds from the corresponding three pots were pooled and homogenized. Oven-dried seeds (moisture ≤ 12%) were equilibrated, loaded into the instrument sample cup as a uniform single layer, and scanned after background calibration with the internal standard. Each sample was loaded and scanned three times as technical replicates, and the mean of the three scans was used as the measurement for that biological replicate. The measured traits included total protein, oil, soluble protein, fiber-related traits, fatty-acid composition, sucrose, and individual amino acids. Protein, oil, and soluble protein contents were reported on a dry-matter basis; moisture content was reported on a wet-matter basis.

### 2.4. DNA Extraction and Amplicon Sequencing

Rhizosphere substrate and root nodules were collected between flowering and the beginning of pod formation. For each cultivar, four biological replicate groups were established (root nodules were three biological replicates), each comprising three pots. Within each replicate group, rhizosphere substrate and root nodules from the three pots were pooled separately to form one composite sample of each material type.

Total DNA was extracted from 0.25 g rhizosphere substrate using the E.Z.N.A.^®^ Soil DNA Kit (D5625; Omega Bio-tek, Norcross, GA, USA). Briefly, the substrate was lysed in 725 μL SLX-Mlus Buffer by high-speed vortexing for 5 min. Clarified lysate was treated sequentially with P2 Buffer and cHTR reagent, mixed with XP1 Buffer, loaded onto a purification column, washed with HBC and DNA washing buffers, dried by centrifugation, and eluted in 60 μL preheated (70 °C) elution buffer. DNA was stored at −20 °C until amplification.

For bacterial profiling, the 16S rRNA gene was amplified with primers 338F (5′-ACTCCTACGGGAGGCAGCAG-3′) and 806R (5′-GGACTACHVGGGTWTCTAAT-3′). Fungal communities were profiled by amplifying the ITS region with ITS1F (5′-CTTGGTCATTTAGAGGAAGTAA-3′) and ITS2R (5′-GCTGCGTTCTTCATCGATGC-3′) (55 °C annealing, 35 cycles). The PCR program recorded in the source protocol was 95 °C for 3 min, followed by cycling at 95 °C for 30 s, 55 °C for 30 s, and 72 °C for 45 s. Bacterial PCR was performed using TransStart FastPfu DNA Polymerase (TransGen Biotech, Beijing, China; Cat. No. AP221-02) on an ABI GeneAmp 9700 thermocycler (Applied Biosystems, Foster City, CA, USA). Each sample was amplified in triplicate, and the three PCR products were pooled, checked by 2% agarose gel electrophoresis, purified with an AxyPrep DNA Gel Extraction Kit (Axygen, Union City, CA, USA), eluted in Tris-HCl, and quantified using the QuantiFluor-ST system (Promega, Madison, WI, USA) before pooling for sequencing. Eight-base sample barcodes were added to the primers. Purified amplicons were sequenced by Majorbio Bio-Pharm Technology Co., Ltd. (Shanghai, China) on an Illumina paired-end PE300 platform (Illumina, San Diego, CA, USA). For bacterial data, paired-end reads were demultiplexed, quality-filtered, merged based on overlap, and denoised to generate ASVs. Fungal ITS reads were processed using DADA2 to generate ASVs. ASVs were used for taxonomic classification and downstream community analyses.

After loosely adhering growth substrate was removed, nodules > 2 mm in diameter were collected aseptically and kept on ice. Nodules were surface sterilized sequentially with sterile water (0.5 min), 75% ethanol (1 min), 2% sodium hypochlorite (3 min), absolute ethanol (1 min), and sterile water (0.5 min). Sterilized nodules were placed on solid YMA medium for 24 h; the absence of colony growth was used as a sterility check. Microbial DNA extracted from surface-sterilized soybean nodules was used for bacterial community profiling. A nested PCR strategy was employed for amplification of bacterial 16S rRNA gene fragments. The first round of amplification was performed using the primer pair 799F–1392R with an annealing temperature of 55 °C for 27 cycles. The second round was performed using primers 799F (5′-AACMGGATTAGATACCCKG-3′) and 1193R (5′-ACGTCATCCCCACCTTCC-3′) with an annealing temperature of 55 °C for 13 cycles.

PCR amplification was performed in a 20 μL reaction mixture containing 10 μL of 2× Pro Taq, 0.8 μL of forward primer (5 μM), 0.8 μL of reverse primer (5 μM), template DNA at approximately 10 ng/μL, and ddH_2_O to a final volume of 20 μL. Amplification was conducted using a T100 Thermal Cycler. The thermal cycling program consisted of an initial denaturation at 95 °C for 3 min, followed by denaturation at 95 °C for 30 s, annealing at 55 °C for 30 s, and extension at 72 °C for 45 s. A final extension was performed at 72 °C for 10 min, followed by holding at 10 °C.

Paired-end raw sequencing reads from nodule samples were quality-filtered using fastp (version 0.23.4). Bases at the ends of reads with quality scores below 20 were trimmed using a 50 bp sliding window, and reads were truncated when the average quality score within the window fell below 20. Reads shorter than 50 bp after quality filtering and reads containing more than five ambiguous bases (N) were discarded. Paired-end reads were subsequently merged using FLASH (version 1.2.11) based on their overlapping regions, with a minimum overlap length of 10 bp and a maximum mismatch ratio of 0.2 within the overlap region. Sequences were demultiplexed according to sample-specific barcodes and primer sequences and reoriented when necessary. No barcode mismatches were permitted, whereas a maximum of two mismatches were allowed in the primer sequences.

Quality-filtered sequences were clustered into operational taxonomic units (OTUs) at 97% sequence identity using USEARCH (version 11), and chimeric sequences were removed during OTU processing. Representative OTU sequences were taxonomically classified using the RDP Classifier (version 2.11) against the SILVA 138.2/16S bacterial reference database with a confidence threshold of 70%. Sequences assigned to chloroplasts and mitochondria were removed prior to downstream community analyses.

### 2.5. Microbial Community and Statistical Analyses

Root-nodule numbers among the six cultivars were compared using one-way ANOVA followed by Tukey’s HSD; values are presented as means ± standard errors. Alpha diversity was evaluated using the Shannon and Sobs indices. Group differences were assessed using Wilcoxon rank-sum tests. Beta diversity was visualized by principal coordinates analysis (PCoA) based on Bray–Curtis dissimilarities, and group differences were tested using ANOSIM with 999 permutations. The ANOSIM statistic (*R*) and permutation *p* value are reported. Genus-level differential abundance was evaluated using Wilcoxon rank-sum tests. Associations between candidate-genus relative abundance and seed protein concentration were assessed by linear regression; the coefficient of determination (*R*^2^) and regression *p* values are reported. Community assembly was assessed at the genus level using the βNTI workflow on the Majorbio platform (R 3.3.1 with vegan), with a prevalence threshold of 0 and 999 randomizations. Values of βNTI < −2 and βNTI > +2 were interpreted as homogeneous and heterogeneous selection, respectively. For comparisons with |βNTI| < 2, Bray–Curtis-based Raup–Crick values (RCBray) were used to partition stochastic processes: RCBray > 0.95 indicated dispersal limitation, RCBray < −0.95 indicated homogenizing dispersal, and |RCBray| < 0.95 indicated undominated processes. The Sloan neutral community model was fitted to the relationship between taxon occurrence frequency and mean relative abundance; model fit (*R*^2^), Nm, and migration rate (m) were reported [35]. Bacterial and fungal co-occurrence networks were constructed separately for the high- and low-protein groups using ggClusterNet::network.pip [36]. ASVs with an abundance lower than 0.02 were excluded, and the remaining ones were included in the analysis; Pearson correlations with |*r*| > 0.8 and unadjusted *p* < 0.05 were retained as edges. Modules were detected using the fast-greedy algorithm, and node size represented degree. Robustness was estimated separately with Robustness.Random.removal (Top = 0, r.threshold = 0.8, *p*.threshold = 0.05, method = Spearman): nodes were removed randomly and the proportion of species remaining was tracked. Vulnerability was calculated from the absolute group-specific correlation matrix as an undirected weighted graph; vertices with degree 0 were removed, and the maximum vertex information centrality was used as the network vulnerability index. For subnetwork, module_display.2 was run with select.mod = “no” and num = 5, so modules with fewer than five ASVs were omitted from the visualization.

Statistical analyses and data visualization were performed using R version 4.5.2 in RStudio. Unless otherwise specified, statistical significance was defined as *p* < 0.05.

Data processing, statistical analysis, network analysis, and visualization were conducted using the R packages ggplot2, vegan, dplyr, impute, WGCNA, data.table, ggrepel, SpiecEasi, and ggClusterNet, as appropriate.

## 3. Results

### 3.1. High-Protein Cultivars Accumulated More Seed Nitrogen, Protein, and Amino Acids

Kjeldahl analysis confirmed a clear nitrogen contrast between the seeds of two cultivar groups. Seed total nitrogen was significantly higher in KD80, KD91, and SN148 than in BD40, KD96, and KD122 (*p* ≤ 0.05). KD91 exceeded BD40 by approximately 7 g·kg^−1^ total nitrogen, whereas differences among cultivars within the same protein class were not significant (Supplementary Figure S1). This group-level separation validates the use of the six cultivars as contrasting material for investigating belowground factors associated with seed nitrogen accumulation.

Near-infrared analysis produced the same phenotype at the level of total seed protein. Protein concentrations were 45.51%, 46.29%, and 43.08% in KD80, KD91, and SN148, respectively, compared with 39.58%, 37.72%, and 36.08% in BD40, KD96, and KD122. Soluble protein followed the same pattern, ranging from 36.5 to 39.2% in the high-protein cultivars and from 28.8 to 31.9% in the low-protein cultivars (Table 2). Most proteinogenic amino acids were also higher in the high-protein group, including glutamate, aspartate, arginine, lysine, leucine, serine, threonine, valine, and isoleucine. In contrast, oil content was lower in the high-protein cultivars (17.73–18.82%) than in the low-protein cultivars (21.29–22.41%), consistent with the well-established inverse relationship between soybean seed protein and oil [1,2,3,4]. Contents of protein, amino acids, oil and other components in grains of different soybean cultivars and tables for protein contents under different accumulated-temperature zones are presented in Supplementary Tables S1 and S2.

### 3.2. High-Protein Soybean Cultivars Exhibited Greater Nodulation, Whereas in the Nodule Bacterial Community No Significant Between-Group Difference Was Detected

**Figure 1:**
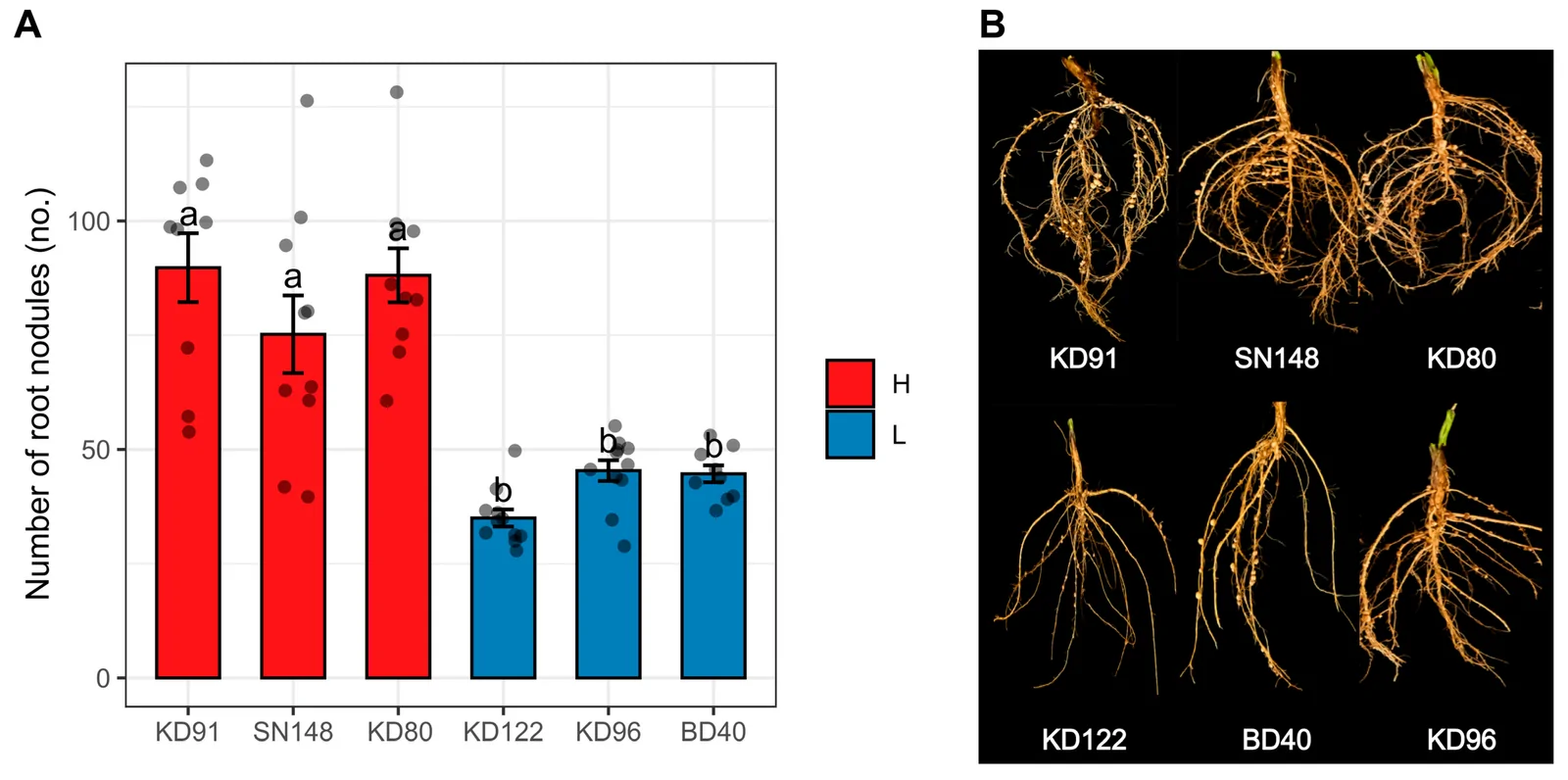
Root nodule counts and representative root/nodule phenotypes in six soybean cultivars. (**A**) Bars show means ± standard errors (SEs), and points show individual observations. Different letters denote significant differences among cultivars (one-way ANOVA with Tukey’s HSD, *p* < 0.05). H and L indicate high- and low-protein cultivars, respectively. (**B**) Representative root systems and nodules from the high-protein cultivars KD91, SN148, and KD80 (top row) and the low-protein cultivars KD122, BD40, and KD96 (bottom row). Images show representative plants grown under the same conditions.

Root nodule number showed a clear and statistically supported contrast among cultivars (Figure 1). KD91, SN148, and KD80, the three high-protein cultivars, were assigned significance group a, whereas KD122, KD96, and BD40, the three low-protein cultivars, were assigned group b. Thus, the high-protein cultivars formed significantly more nodules than the low-protein cultivars (*p* < 0.05), while no significant differences were detected within either protein group. This result establishes enhanced nodulation as a phenotype associated with the high-protein group. Because nodule biomass and nitrogenase activity were not measured, however, a greater nodule number should not be interpreted as direct evidence of greater biological nitrogen fixation. Representative root and nodule phenotypes of the six cultivars are shown in Figure 1B.

**Figure 2:**
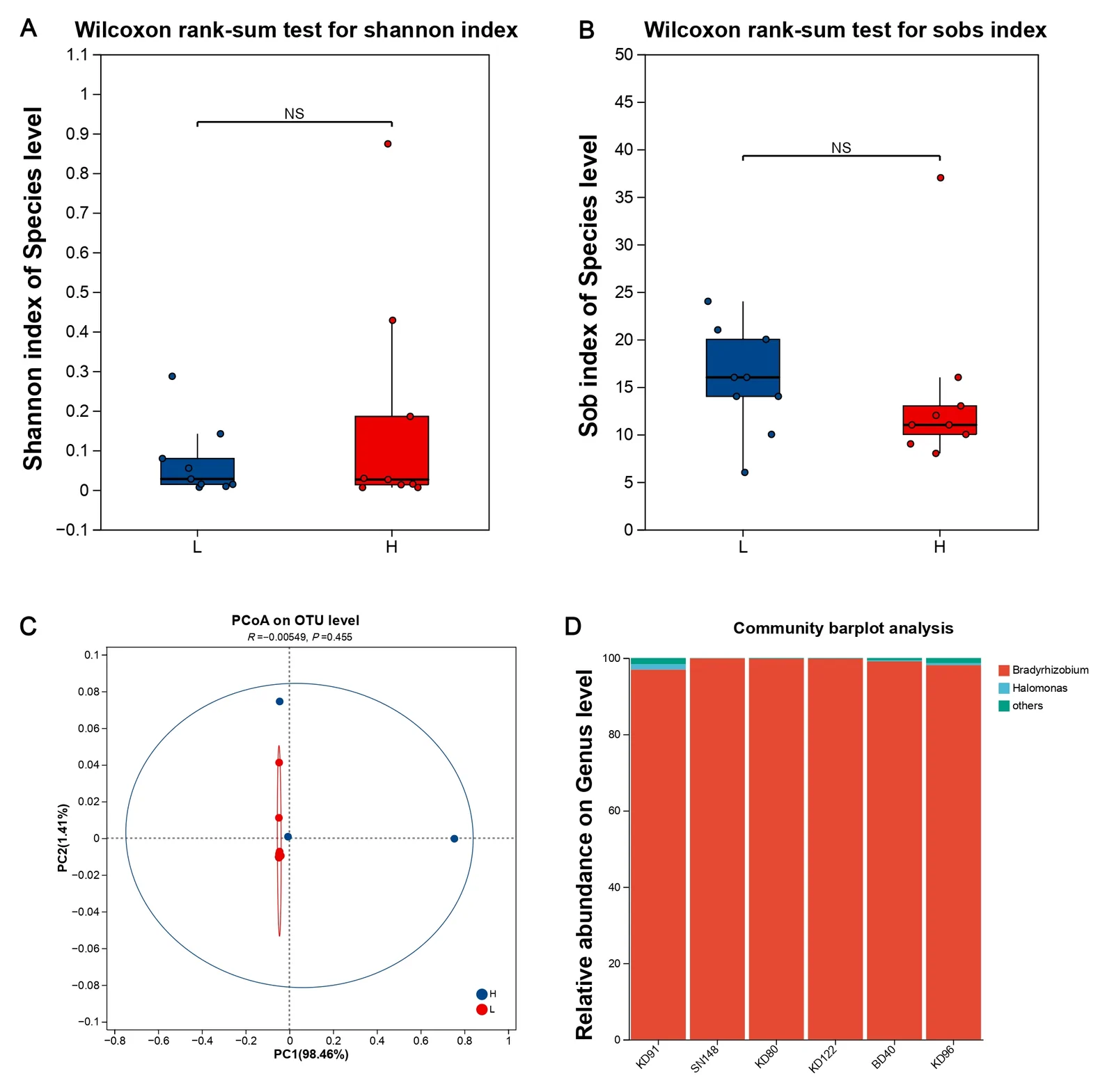
Bacterial community structure in soybean root nodules. (**A**) Shannon diversity and (**B**) Sobs richness between low-protein (L) and high-protein (H) groups; NS, not significant. (**C**) PCoA at the OTU level; ANOSIM (999 permutations) yielded *R* = −0.00549, *p* = 0.455. (**D**) Genus-level relative abundance, showing strong dominance of *Bradyrhizobium* across cultivars. Blue and red points represent samples from the high-protein (H) and low-protein (L) groups, respectively, and the corresponding ellipses indicate the 95% confidence regions.

Despite the pronounced difference in nodule number, the bacterial community inside nodules was comparatively conserved (Figure 2). Neither Shannon diversity nor Sobs richness differed significantly between the high- and low-protein groups. ANOSIM of Bray–Curtis dissimilarities detected no significant group separation (*R* = −0.00549, *p* = 0.455). At the genus level, *Bradyrhizobium* overwhelmingly dominated all nodule samples, with *Halomonas* and other genera representing only minor fractions. Thus, the high-protein phenotype was associated with the extent of nodulation but not with broad taxonomic restructuring of the nodule bacterial community, directing attention to the more diverse rhizosphere compartment.

### 3.3. High- and Low-Protein Soybeans Harbored Distinct Rhizosphere Microbial Communities

**Figure 3:**
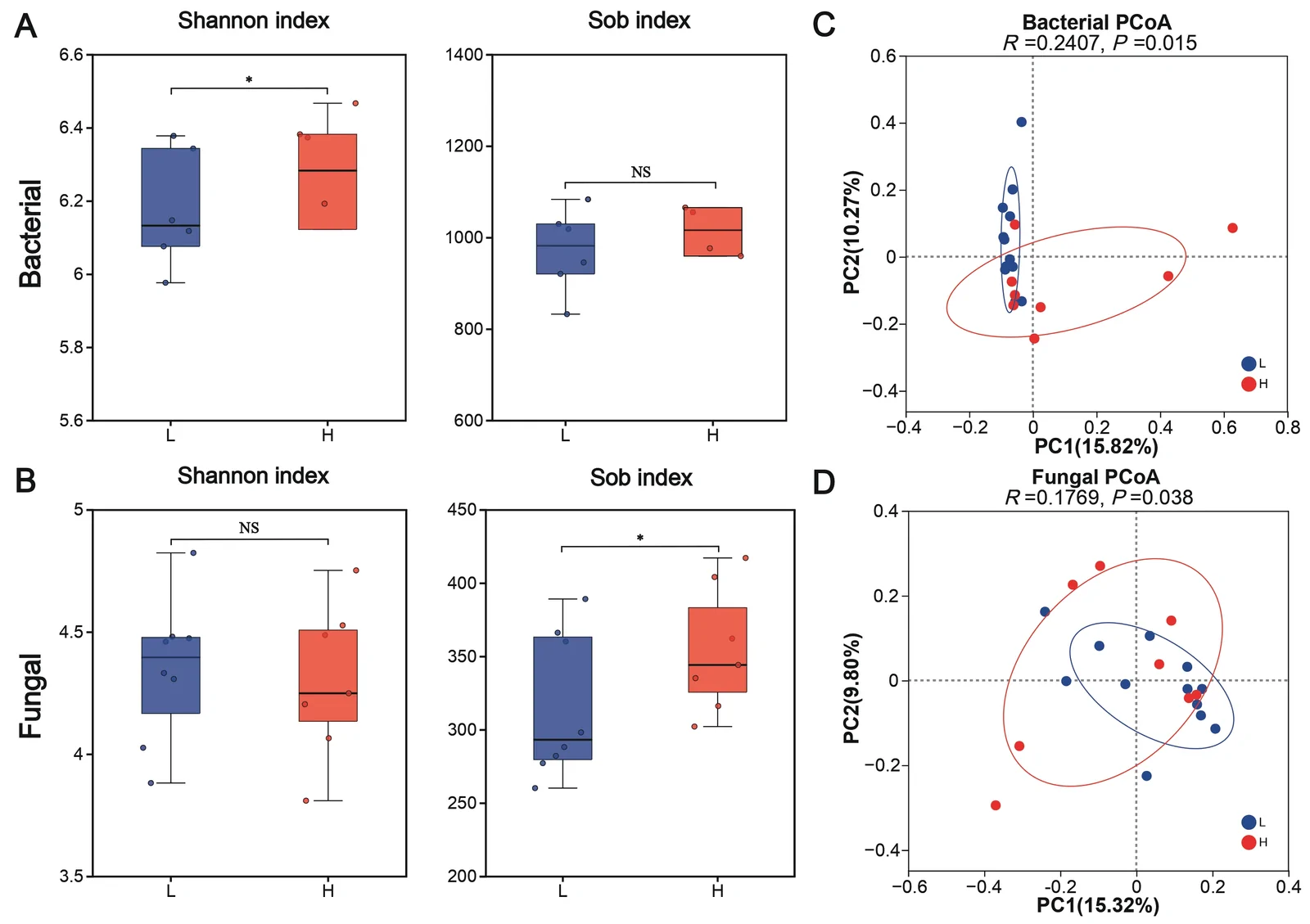
Rhizosphere bacterial and fungal alpha and beta diversity in low-protein (L) and high-protein (H) soybeans. (**A**) Bacterial Shannon and Sobs indices; (**B**) fungal Shannon and Sobs indices; (**C**) bacterial PCoA and (**D**) fungal PCoA, with ANOSIM results (999 permutations) of *R* = 0.2407, *p* = 0.015 and *R* = 0.1769, *p* = 0.038, respectively. * indicates *p* < 0.05; NS, not significant. Blue and red points represent samples from the high-protein (H) and low-protein (L) groups, respectively, and the corresponding ellipses indicate the 95% confidence regions.

Rhizosphere communities showed substantially stronger differentiation between protein groups than did nodule communities (Figure 3). For bacteria, Shannon diversity differed significantly between the low- and high-protein groups, whereas Sobs richness did not. ANOSIM of Bray–Curtis dissimilarities indicated significant bacterial community separation (*R* = 0.2407, *p* = 0.015). For fungi, Shannon diversity was not significantly different, whereas Sobs richness differed significantly; ANOSIM likewise indicated significant fungal community separation (*R* = 0.1769, *p* = 0.038). These results indicate that contrasting seed protein phenotypes were accompanied by coordinated changes in both bacterial and fungal rhizosphere communities, although the diversity metrics affected differed between kingdoms.

### 3.4. Specific Rhizosphere Taxa Had Higher Relative Abundance in the High-Protein Group

**Figure 4:**
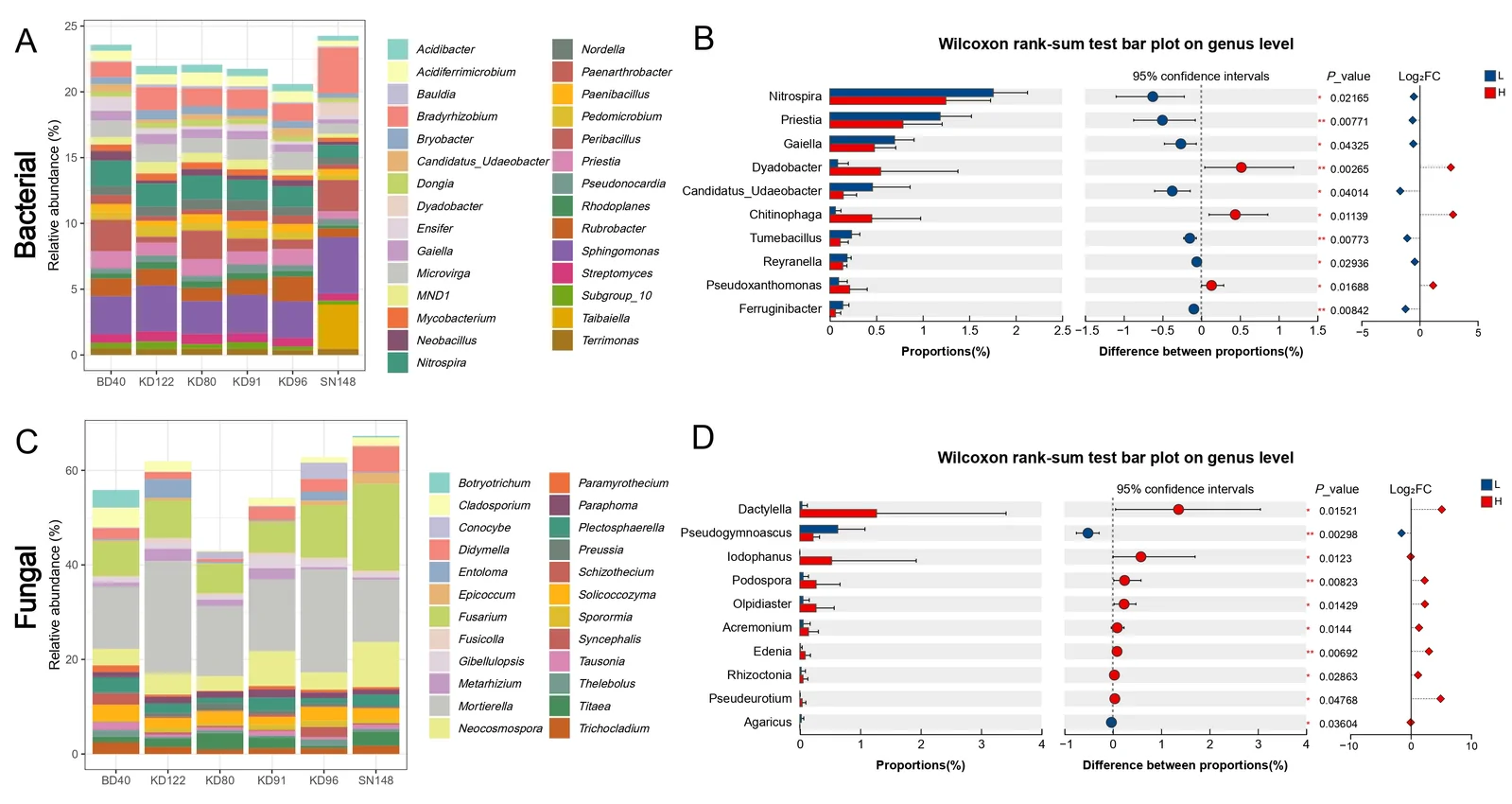
Rhizosphere taxonomic composition and genus-level differential abundance. (**A**,**B**) Relative abundance of major bacterial and fungal genera across the six soybean cultivars; (**C**,**D**) Wilcoxon rank-sum comparisons between low-protein (L) and high-protein (H) groups at the genus level. Horizontal intervals indicate 95% confidence intervals.

At the genus level, the six cultivars shared many dominant bacterial and fungal taxa, but the relative abundance of individual genera varied among cultivars and between protein groups (Figure 4A,B). Wilcoxon rank-sum analysis identified ten bacterial genera that differed significantly between the high- and low-protein groups (Figure 4C). *Dyadobacter*, *Chitinophaga*, and *Pseudoxanthomonas* were significantly enriched in the rhizosphere of high-protein soybeans (*p* = 0.00265, 0.01139, and 0.01688, respectively), whereas *Nitrospira*, *Priestia*, *Gaiella*, *Candidatus_Udaeobacter*, *Tumebacillus*, *Reyranella*, and *Ferruginibacter* were more abundant in the low-protein group (*p* < 0.05). Significant differences were also detected for several fungal genera, including *Dactylella*, *Pseudogymnoascus*, *Iodophanus*, *Podospora*, *Olpidiaster*, *Acremonium*, *Edenia*, *Rhizoctonia*, *Pseudeurotium*, and *Agaricus* (Figure 4D; *p* < 0.05). Because the subsequent phenotype-association analysis focused on bacteria, *Dyadobacter*, *Pseudoxanthomonas*, and *Chitinophaga* were carried forward as candidate high-protein-associated bacterial taxa for network and regression analyses.

### 3.5. Contrasting Protein Phenotypes Were Associated with Altered Rhizosphere Community Assembly

**Figure 5:**
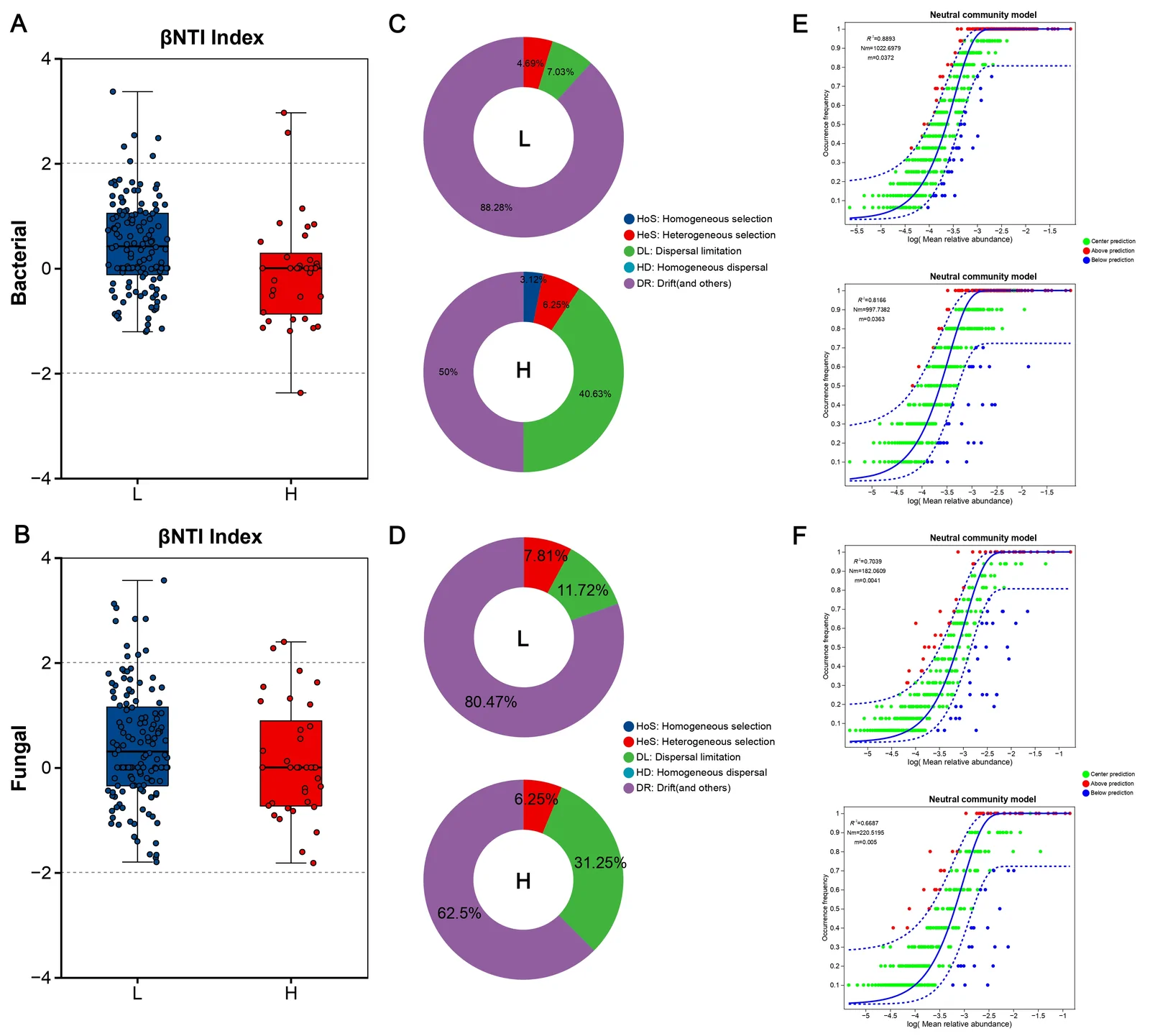
Ecological assembly of bacterial and fungal rhizosphere communities. (**A**,**B**) βNTI distributions; (**C**,**D**) contributions of homogeneous selection, heterogeneous selection, dispersal limitation, homogenizing dispersal, and drift/other undominated processes; (**E**,**F**) neutral community model fits.

Null-model analysis revealed a marked shift in rhizosphere community assembly between protein groups (Figure 5). For bacteria, drift/undominated processes decreased from 88.28% in L to 50.00% in H, whereas dispersal limitation increased from 7.03% to 40.63%; heterogeneous selection contributed 4.69% in L and 6.25% in H, with homogeneous selection contributing 3.13% in H. For fungi, dispersal limitation increased from 11.72% in L to 31.25% in H, while drift/undominated processes declined from 80.47% to 62.50%. Neutral community models fitted bacterial communities with R^2^ values of 0.8893 and 0.8166 and fungal communities with *R*^2^ values of 0.7039 and 0.6687. These results indicate stronger dispersal constraints in the high-protein rhizosphere.

### 3.6. Co-Occurrence Networks Further Prioritized High-Protein-Associated Bacterial Taxa

**Figure 6:**
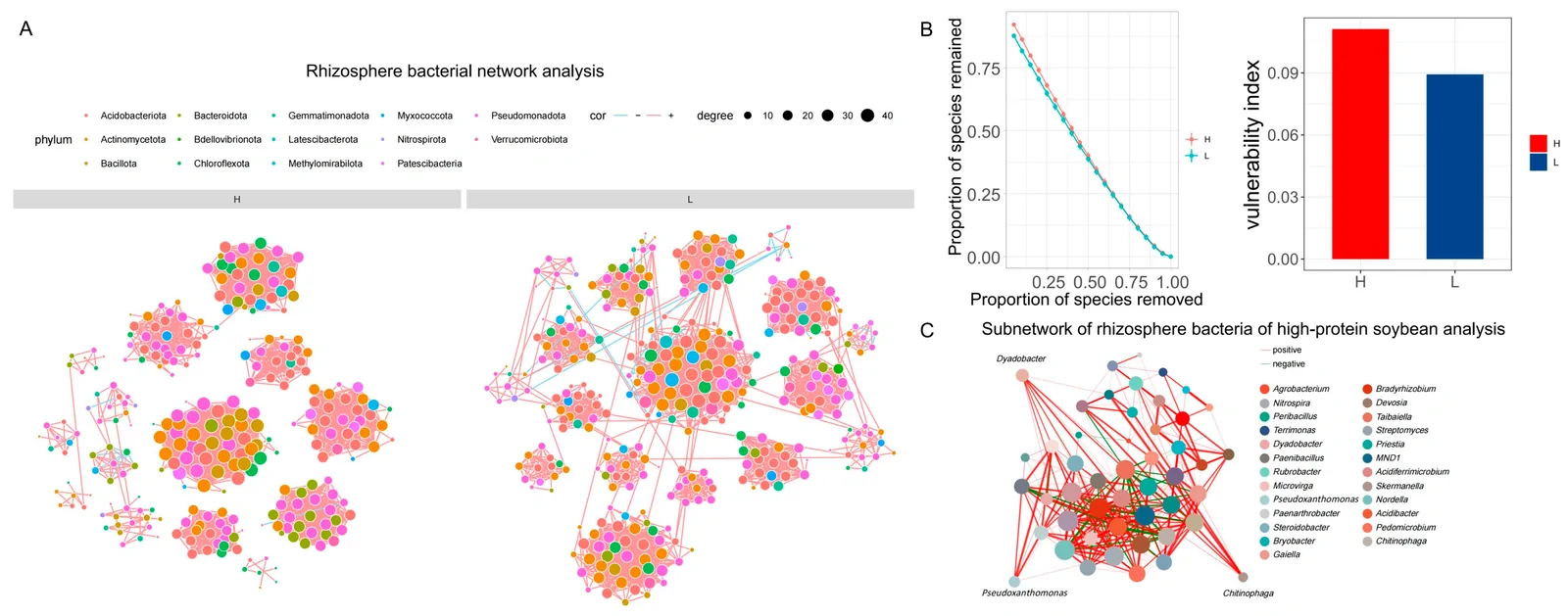
Rhizosphere bacterial co-occurrence network analysis. (**A**) Network topology of high-protein (H) and low-protein (L) rhizospheres. (**B**) Robustness curves and vulnerability index under simulated species removal. (**C**) High-protein bacterial subnetwork highlighting *Dyadobacter*, *Pseudoxanthomonas*, and *Chitinophaga*.

**Figure 7:**
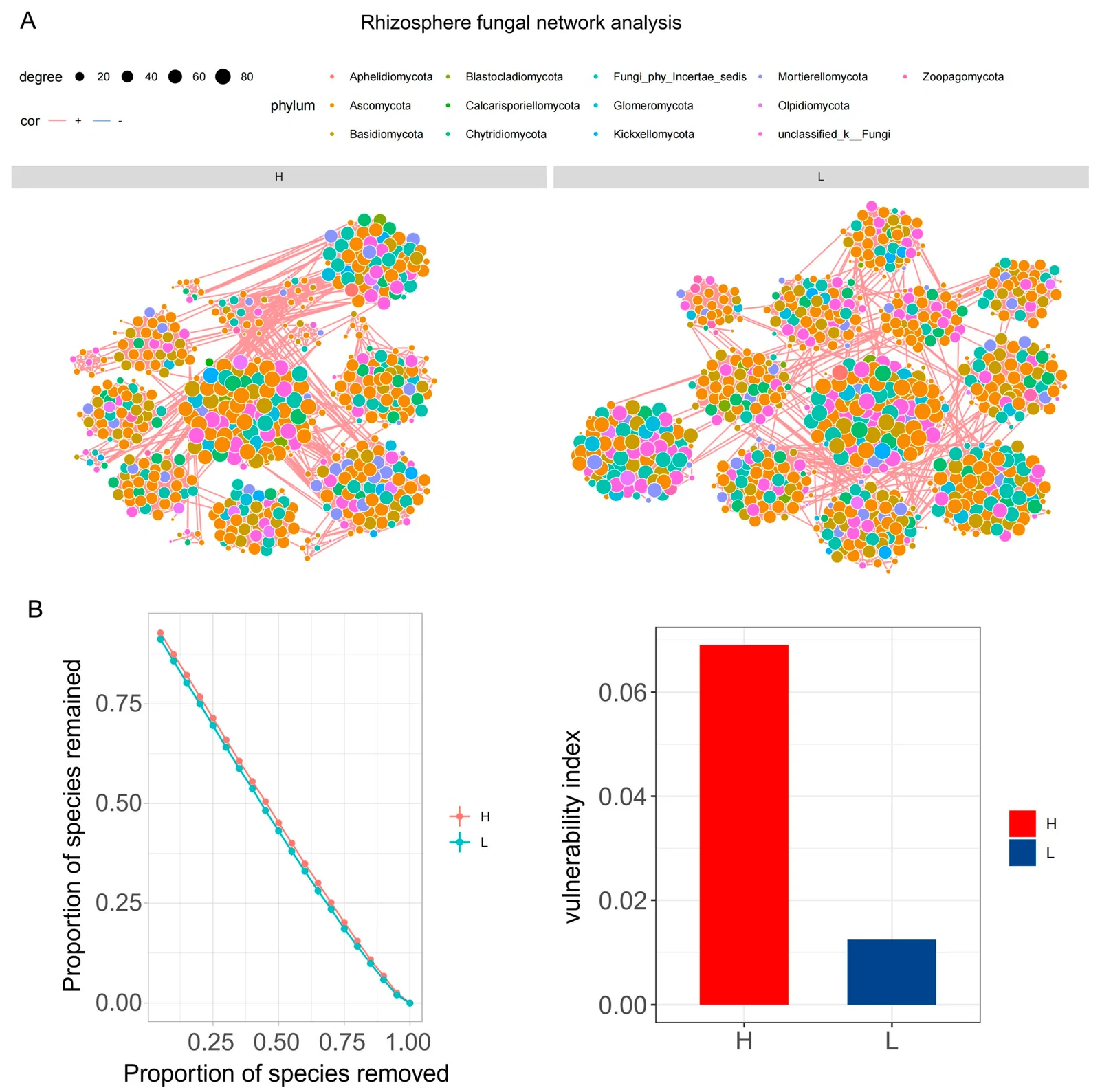
Rhizosphere fungal co-occurrence network analysis in high-protein (H) and low-protein (L) soybean groups. (A) Rhizosphere fungal co-occurrence networks in the H (left) and L (right) groups. (B) Network robustness (left) and vulnerability index (right) under simulated species removal.

Co-occurrence network analysis revealed visually distinct bacterial and fungal interaction architectures between protein groups. In the bacterial network (Figure 6A,B), the high-protein group exhibited greater network vulnerability than the low-protein group, indicating that removal of influential nodes may have a larger effect on network integrity. The three candidate genera were first shortlisted because they had higher genus-level relative abundance in the high-protein group and nominal Wilcoxon *p* values < 0.05; they were then highlighted in the high-protein bacterial network display, without an additional degree or centrality cutoff (Figure 6C). Fungal networks also differed in topology and taxonomic connectivity between groups. Because co-occurrence edges represent statistical associations rather than direct ecological interactions, these networks should be interpreted as hypotheses about community organization. Nonetheless, the network results reinforce the conclusion from beta diversity and assembly analyses that the high- and low-protein rhizospheres represent different ecological states. The topological properties of the bacterial and fungal co-occurrence networks are summarized in Supplementary Table S3. Fungal networks, network robustness, and vulnerability are shown in (Figure 7).

### 3.7. Dyadobacter and Pseudoxanthomonas Were Positively Associated with Seed Protein Content

**Figure 8:**
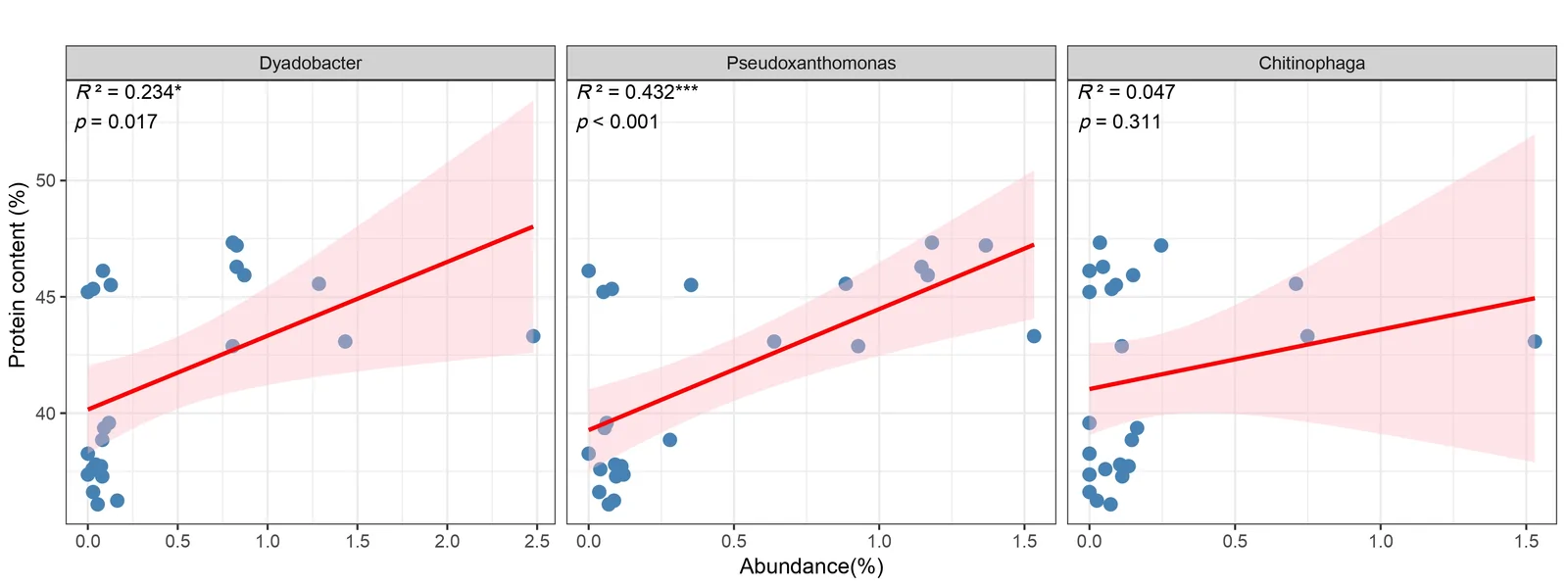
Each point represents one biological replicate, consisting of a composite sample from three pots. The relative abundance of each genus in a rhizosphere sample was paired with the seed protein content measured from seeds collected from the corresponding replicate group. Four biological replicates were included for each cultivar (*n* = 24 in total). Relationships between seed protein concentration and rhizosphere relative abundance of *Dyadobacter*, *Pseudoxanthomonas*, and *Chitinophaga*. *Dyadobacter* (*R*^2^ = 0.234, *p* = 0.017) and *Pseudoxanthomonas* (*R*^2^= 0.432, *p* < 0.001) are significant; *Chitinophaga* is not significant (*R*^2^ = 0.047, *p* = 0.311).

To test whether the candidate bacteria identified by differential abundance and network analyses tracked seed protein concentration across samples, genus abundance was regressed against seed protein content using 24 matched sample pairs (Figure 8). *Dyadobacter* abundance was positively associated with protein content (*R*^2^= 0.234, *p* = 0.017), and *Pseudoxanthomonas* showed a stronger positive relationship (*R*^2^= 0.432, *p* < 0.001). In contrast, *Chitinophaga* was not significantly associated with protein content (*R*^2^= 0.047, *p* = 0.311). These results refine the initial three-taxon candidate set: *Dyadobacter* and *Pseudoxanthomonas* emerged as the strongest protein-associated candidates, whereas *Chitinophaga* remained an exploratory candidate whose enrichment in the high-protein rhizosphere was not accompanied by a significant across-sample protein relationship.

## 4. Discussion

The clearest physiological distinction between the two soybean groups was their nitrogen and protein status. High-protein cultivars accumulated more seed total nitrogen, total protein, soluble protein, and most measured amino acids, while showing lower oil concentrations. This pattern is consistent with the central role of nitrogen supply in soybean storage-protein accumulation and with the well-established negative relationship between seed protein and oil in many soybean genetic backgrounds [1,2,3,4,5].

Higher nodule numbers co-occurred with higher seed nitrogen and protein concentrations in the high-protein cultivars. Soybean depends strongly on symbiotic nitrogen fixation, and effective nodulation can support plant nitrogen acquisition [6,7,8,9,10,11,12,14,15]. Nevertheless, nodule number is not equivalent to nitrogen-fixation capacity: nodule size, activity, occupancy, carbon supply, and symbiotic efficiency all affect the amount of fixed nitrogen delivered to the plant. Accordingly, our data support an association between enhanced nodulation and high seed protein rather than a causal claim that additional nodules produced the protein difference. Although seed total nitrogen concentration was measured, the contribution of biological nitrogen was not quantified. Measurements of nodule biomass, nitrogenase activity, and ^15^N-derived nitrogen would be required to test this mechanism directly.

A clear contrast emerged between nodule number and nodule bacterial community profiles. Although high-protein cultivars had more nodules, no statistically significant between-protein-group differences were detected in nodule bacterial alpha diversity or overall community structure, and *Bradyrhizobium* dominated across cultivars. This pattern is consistent with the strong host filtering imposed by the nodule environment and with previous soybean studies showing *Bradyrhizobium* as a dominant nodule inhabitant [13]. The absence of a detected group-level difference does not rule out strain-level variation in symbiotic performance, which genus-level amplicon profiling cannot resolve.

In contrast, the rhizosphere displayed significant bacterial and fungal community differentiation. This compartment contrast suggests that the ecological signature associated with seed protein phenotype is expressed more strongly in the taxonomically diverse rhizosphere than in the highly selective nodule interior. Soybean genotype is known to contribute to rhizosphere microbiome assembly [16,17], and the present results are consistent with genotype-dependent recruitment or filtering. Because the study compares six distinct cultivars rather than near-isogenic lines, however, protein class remains partially confounded with genetic background.

In genus-level comparisons, *Dyadobacter*, *Pseudoxanthomonas*, and *Chitinophaga* had higher relative abundance in high-protein rhizospheres, with nominal group differences based on unadjusted *p* values < 0.05. These genera were carried forward as candidate taxa for network and regression analyses. This pattern is consistent with the study of Han et al. [31], which linked soybean oil-related quality traits to rhizosphere microbiome composition and then demonstrated an effect of Massilia inoculation on seed oil content. The present results provide evidence of association between cultivar group, microbial relative abundance, and seed protein, but do not establish selective recruitment or a causal microbial effect. Inoculation experiments are needed to test whether representative strains alter seed composition.

These associations may reflect host genotype or physiology, root exudation, root architecture, or nutrient status shaping microbial niches; microorganisms may also affect nutrient acquisition or plant metabolism. The present design cannot establish the direction or magnitude of these relationships, so functional experiments are needed to distinguish among these possibilities.

This distinction is important because enrichment can arise either because a microorganism contributes to the host phenotype or because the host phenotype, root exudation pattern, root architecture, or nutrient status creates a niche favorable to that microorganism. The current results therefore support a bidirectional working model in which soybean genotype and protein-related physiology shape rhizosphere recruitment, while selected microorganisms may in turn influence nutrient acquisition or plant metabolism. Functional experiments are needed to resolve the direction and magnitude of these effects.

*Pseudoxanthomonas* showed the strongest relationship with seed protein content (*R*^2^ = 0.432, *p* < 0.001). Plant-associated *Pseudoxanthomonas* strains have been reported to possess growth-promoting traits, including indole-related compound production, siderophore production, and stress-alleviation capacity, and experimental inoculation of some strains has improved plant growth under normal or saline conditions. These observations make *Pseudoxanthomonas* a biologically plausible candidate, but such functions cannot be assigned to the soybean-associated amplicon sequence variants detected here without isolation and strain-level testing.

*Dyadobacter* was also positively associated with seed protein content (*R*^2^ = 0.234, *p* = 0.017). Members of *Dyadobacter* (Bacteroidota) occur in soil and plant-associated habitats, and some isolates have displayed plant growth-promoting traits, including diazotrophic potential or associations with improved plant performance. The moderate amount of protein variation explained by the regression indicates that *Dyadobacter* should be viewed as a candidate biomarker or potentially functional taxon rather than a proven determinant of seed protein.

*Chitinophaga* provides a useful internal contrast. It was significantly enriched in the high-protein rhizosphere and was retained through network screening, but its abundance was not significantly related to protein content across samples (*R*^2^ = 0.047, *p* = 0.311). Thus, *Chitinophaga* may reflect the broader microbial environment associated with high-protein cultivars, may act through interactions with other community members, or may simply co-respond to host filtering. It should be retained as a secondary candidate for culture-based screening but not presented as statistically validated evidence of a protein-promoting bacterium.

The community assembly analyses indicate that the two protein groups differed not only in taxonomic composition but also in the inferred processes structuring their rhizosphere communities. Dispersal limitation increased from 7.03% in low-protein to 40.63% in high-protein bacterial communities and from 11.72% to 31.25% in fungal communities, while drift/undominated processes declined. Neutral community model fits were also lower in the high-protein group for both bacteria and fungi. Together, these patterns are consistent with altered ecological filtering and spatial organization in the high-protein rhizosphere, although null-model results should be interpreted as process-level hypotheses rather than direct measurements of mechanisms [32,33].

Network analysis revealed distinct topologies and a higher vulnerability index in the high-protein bacterial network. Higher vulnerability may indicate greater sensitivity of network connectivity to node removal; by itself, it does not imply reduced ecological health or establish that particular taxa are keystone species. The high-protein network places the three differentially abundant candidate genera in community context. Because differential-abundance, network, and regression analyses used the same 24 matched samples, network results provide supporting evidence rather than independent validation. Network edges represent correlations rather than direct interactions, and synthetic-community or inoculation experiments are needed to determine whether these taxa interact with one another or with *Bradyrhizobium*.

Several limitations define the scope of the conclusions. First, the study contains six cultivars, so protein class is partially confounded with genotype; larger diversity panels or near-isogenic materials would be required to isolate protein phenotype from unrelated genetic effects. Second, the microbiome results are associative and do not establish microbial causality. Third, nodule function was inferred from nodule number rather than measured directly. Fourth, genus-level amplicon sequencing cannot establish strain identity or function.

A direct next step is to isolate representative *Pseudoxanthomonas* and *Dyadobacter* strains, together with *Chitinophaga* as a secondary candidate, from contrasting soybean rhizospheres and characterize traits relevant to nitrogen and nutrient acquisition, including nitrogen-fixation potential, phosphate solubilization, siderophore production, indole-related compounds, ACC deaminase activity, and effects on *Bradyrhizobium* growth. Single-strain and co-inoculation experiments with *Bradyrhizobium* should then quantify nodule number and biomass, nitrogenase activity, plant and seed nitrogen, seed protein and oil, amino-acid profiles, and expression of nitrogen-assimilation and storage-protein genes. Such experiments would test whether the associations identified here represent functional contributions to soybean protein accumulation, analogous in logic to the functional validation used for *Massilia* and soybean oil [31].

## 5. Conclusions

Among the six soybean cultivars examined, cultivar groups with contrasting seed protein contents differed in nodulation and rhizosphere microbial characteristics. High-protein cultivars formed significantly more root nodules, but their nodule bacterial communities remained compositionally similar to those of low-protein cultivars and were dominated by *Bradyrhizobium*. In contrast, rhizosphere bacterial and fungal communities differed significantly between protein groups in community structure, taxonomic composition, assembly processes, and network organization. *Dyadobacter*, *Pseudoxanthomonas*, and *Chitinophaga* were enriched in high-protein rhizospheres and prioritized by network analysis; among them, *Dyadobacter* and *Pseudoxanthomonas* showed significant positive associations with seed protein content, whereas *Chitinophaga* did not. Seed protein phenotype and cultivar genetic background are confounded in this design, so the observed microbial differences cannot be attributed to protein phenotype alone. These associations also do not establish that the candidate taxa increased seed protein. Strain inoculation, nitrogen-fixation measurements, and validation across additional substrates and field conditions are needed to test these hypotheses.

## Figures and Tables

**Table 1:** Physicochemical properties of the peat-based growth substrate used for soybean cultivation.

Substrate Property	Value
pH	8.53 ± 0.08
Total nitrogen (%)	0.0889 ± 0.0020
Total phosphorus (%)	0.0644 ± 0.0006
Total potassium (%)	0.6462 ± 0.0060
Hydrolyzable nitrogen (mg·kg^−1^)	43.53 ± 1.04
Available phosphorus (mg·kg^−1^)	12.28 ± 0.25
Available potassium (mg·kg^−1^)	78.88 ± 2.12
Organic matter (g·kg^−1^)	17.28 ± 0.28

Note: Values are mean ± SD of three analytical replicates (*n* = 3).

**Table 2:** Selected seed composition traits for the six focal cultivars.

Cultivar	Group	Protein (% Dry Basis)	Oil (% Dry Basis)	Soluble Protein (% Dry Basis)
BD40	L	39.31 ± 0.38	21.67 ± 0.33	31.63 ± 0.39
KD96	L	37.72 ± 0.00	21.79 ± 0.70	31.21 ± 0.01
KD122	L	36.24 ± 0.22	22.81 ± 0.57	28.76 ± 0.06
KD80	H	45.90 ± 0.55	17.71 ± 0.04	33.16 ± 0.08
KD91	H	46.09 ± 0.29	18.32 ± 0.06	39.40 ± 0.28
SN148	H	43.07 ± 0.02	18.70 ± 0.18	36.48 ± 0.03

## Data Availability

The raw sequence reads generated in this study have been deposited in the NCBI Sequence Read Archive (SRA) under BioProject accession number PRJNA1520118. Reviewers can access the data via the following private link: https://dataview.ncbi.nlm.nih.gov/object/PRJNA1520118?reviewer=5mo803h13jl5kv88kcdnlhn906URL (accessed on 2 September 2026).

## Supplementary Materials

The following supporting information can be downloaded at: https://www.mdpi.com/article/10.3390/microorganisms14102266/s1, Figure S1: Seed total nitrogen concentration in six soybean cultivars; Figure S2: Representative root and nodule phenotypes of high- and low-protein soybean cultivars; Table S1: Seed quality composition of three low-protein and three high-protein soybean cultivars; Table S2: Seed quality traits of soybean cultivar KD91 collected from different locations in Heilongjiang Province, China; Table S3: Topological properties of rhizosphere bacterial and fungal co-occurrence networks in high- and low-protein soybean groups.

## Abbreviations

The following abbreviations are used in this manuscript:

ANOVA	Analysis of variance
L	Low-protein soybean group
H	High-protein soybean group
KD91	Soybean cultivar name Ke dou 91
SN148	Soybean cultivar name Sui nong 148
BD40	Soybean cultivar name Bei dou 40
KD80	Soybean cultivar name Ke dou 80
KD122	Soybean cultivar name Ke dou 122
KD96	Soybean cultivar name Ke dou 96
