# Supplementary material for: Contrasting Seed Protein Phenotypes Are Associated with Nodulation and Rhizosphere Microbiome Assembly in Soybean
Source: Microorganisms. 2026 Oct 5;14(10):2266. doi: 10.3390/microorganisms14102266 (PMC13707409; doi:10.3390/microorganisms14102266)
Supplement: Supplementary file 1 [file microorganisms-14-02266-s001.zip › Supplementary Table S1.pdf]

Table S1. Seed quality composition of three low-protein and three high-protein soybean cultivars.

Component	BD40	KD96	KD122	KD80	KD91	SN148
Moisture (%)	9.08 ± 0.01	10.38 ± 0.40	7.44 ± 0.01	11.20 ± 0.78	9.36 ± 0.35	11.04 ± 0.07
Protein (%)	39.31 ± 0.38	37.72 ± 0.00	36.24 ± 0.22	39.87 ± 0.81	46.09 ± 0.29	43.07 ± 0.02
Ash (%)	5.60	5.43	5.37	5.40	5.67	5.65
Oil (%)	21.67 ± 0.33	21.79 ± 0.70	22.81 ± 0.57	19.98 ± 0.78	18.32 ± 0.06	18.70 ± 0.18
Starch (%)	0.30 ± 0.06	-1.07 ± 0.06	1.31 ± 0.06	-1.40 ± 0.26	-1.54 ± 0.16	-3.28 ± 0.10
Fiber (%)	6.44 ± 0.14	6.45 ± 0.06	6.92 ± 0.04	6.15 ± 0.08	5.94 ± 0.08	5.84 ± 0.14
Water-soluble protein (%)	31.63 ± 0.39	31.21 ± 0.01	28.76 ± 0.06	33.16 ± 0.08	39.40 ± 0.28	36.48 ± 0.03
Oleic acid (%)	28.16 ± 0.08	27.79 ± 0.07	24.49 ± 0.08	23.07 ± 0.01	25.88 ± 0.11	24.90 ± 0.04
Sucrose (%)	5.38 ± 0.03	5.72 ± 0.09	7.30 ± 0.01	6.04 ± 0.01	4.40 ± 0.04	5.53 ± 0.11
Cysteine (%)	0.52 ± 0.01	0.59 ± 0.08	0.43 ± 0.09	0.55 ± 0.04	0.60 ± 0.04	0.55 ± 0.01
Phenylalanine (%)	2.06 ± 0.06	1.92 ± 0.06	1.85 ± 0.04	2.02 ± 0.03	2.34 ± 0.04	2.21 ± 0.10
Alanine (%)	1.55 ± 0.17	1.63 ± 0.03	1.45 ± 0.18	1.67 ± 0.01	1.75 ± 0.18	1.75 ± 0.01
Methionine (%)	0.56 ± 0.01	0.55 ± 0.01	0.56 ± 0.02	0.55 ± 0.01	0.64 ± 0.02	0.58 ± 0.01
Glycine (%)	1.69 ± 0.02	1.58 ± 0.08	1.60 ± 0.04	1.67 ± 0.04	1.89 ± 0.01	1.75 ± 0.01
Glutamic acid (%)	6.84 ± 0.04	6.73 ± 0.04	6.34 ± 0.06	6.76 ± 0.01	8.17 ± 0.02	7.27 ± 0.01
Arginine (%)	2.86 ± 0.02	2.82 ± 0.02	2.55 ± 0.04	2.83 ± 0.02	3.44 ± 0.03	3.13 ± 0.04
Lysine (%)	2.58 ± 0.01	2.53 ± 0.01	2.43 ± 0.03	2.54 ± 0.01	2.94 ± 0.01	2.73 ± 0.04
Tyrosine (%)	1.45 ± 0.04	1.42 ± 0.02	1.36 ± 0.03	1.37 ± 0.02	1.61 ± 0.01	1.53 ± 0.04
Leucine (%)	3.03 ± 0.04	2.95 ± 0.01	2.83 ± 0.03	2.95 ± 0.04	3.48 ± 0.01	3.23 ± 0.01
Taurine (%)	0.06 ± 0.00	0.05 ± 0.01	0.05 ± 0.00	0.03 ± 0.00	0.04 ± 0.00	0.05 ± 0.00
Proline (%)	1.80 ± 0.08	1.76 ± 0.01	1.64 ± 0.08	1.73 ± 0.06	2.07 ± 0.02	1.92 ± 0.01
Tryptophan (%)	0.45 ± 0.03	0.47 ± 0.04	0.39 ± 0.01	0.46 ± 0.04	0.47 ± 0.02	0.48 ± 0.04
Serine (%)	1.61 ± 0.10	1.61 ± 0.04	1.51 ± 0.04	1.59 ± 0.02	1.92 ± 0.04	1.74 ± 0.06
Threonine (%)	1.51 ± 0.02	1.47 ± 0.01	1.44 ± 0.02	1.42 ± 0.06	1.62 ± 0.08	1.56 ± 0.01
Acid detergent fiber (%)	15.90 ± 0.01	16.20 ± 0.11	15.80 ± 0.03	15.73 ± 0.08	16.15 ± 0.01	15.32 ± 0.11
Aspartic acid (%)	4.44 ± 0.02	4.39 ± 0.04	4.14 ± 0.02	4.41 ± 0.04	5.16 ± 0.04	4.64 ± 0.06
Valine (%)	2.05 ± 0.06	1.94 ± 0.04	1.79 ± 0.11	2.03 ± 0.02	2.30 ± 0.04	2.15 ± 0.03
Linolenic acid (%)	7.41 ± 0.04	8.34 ± 0.06	6.44 ± 0.04	7.63 ± 0.08	7.39 ± 0.04	9.26 ± 0.01
Linoleic acid (%)	49.16 ± 0.02	48.84 ± 0.06	56.50 ± 0.03	51.12 ± 0.06	53.24 ± 0.01	51.30 ± 0.03
Isoleucine (%)	1.83 ± 0.08	1.86 ± 0.02	1.70 ± 0.11	1.84 ± 0.06	2.15 ± 0.06	1.95 ± 0.04
Stearic acid (%)	3.33 ± 0.01	3.47 ± 0.01	2.84 ± 0.05	3.27 ± 0.07	3.25 ± 0.08	3.33 ± 0.06
Neutral detergent fiber (%)	16.63 ± 0.01	16.26 ± 0.01	17.45 ± 0.10	15.85 ± 0.04	15.70 ± 0.08	15.17 ± 0.04
Palmitic acid (%)	9.93 ± 0.03	10.33 ± 0.04	9.28 ± 0.01	10.55 ± 0.06	10.56 ± 0.04	10.35 ± 0.01
Histidine (%)	1.06 ± 0.04	1.08 ± 0.08	0.99 ± 0.03	1.06 ± 0.06	1.15 ± 0.01	1.13 ± 0.04
Eicosenoic acid (%)	0.42 ± 0.02	0.35 ± 0.04	0.35 ± 0.04	0.37 ± 0.03	0.44 ± 0.04	0.46 ± 0.06

Note: Values are mean ± sample SD of two DA 7250 measurements per cultivar, Moisture is reported on a wet basis; all other components are reported on a dry basis. BD40, KD96, and KD122 are low-protein cultivars; KD80, KD91, and SN148 are high-protein cultivars.
