# Supplementary material for: Contrasting Seed Protein Phenotypes Are Associated with Nodulation and Rhizosphere Microbiome Assembly in Soybean
Source: Microorganisms. 2026 Oct 5;14(10):2266. doi: 10.3390/microorganisms14102266 (PMC13707409; doi:10.3390/microorganisms14102266)
Supplement: Supplementary file 1 [file microorganisms-14-02266-s001.zip › Supplementary Table S2.pdf]

Table S2. Seed quality traits of soybean cultivar KD91 collected from different locations in Heilongjiang Province, China.

Sampling location	Moisture (%, wet basis)	Protein (%, dry basis)	Oil (%, dry basis)	Water-soluble protein (%, dry basis)	Accumulated temperature zone
KD91–Heilongjiang Academy of Agricultural Sciences, Harbin	5.78	45.11	18.99	36.4	First accumulated temperature zone
KD91–Dahai Seed Industry, Kedong	4.9	45.59	17.24	36.6	Fourth accumulated temperature zone
KD91–Daqing Branch	4.73	45.03	19.05	36.5	First accumulated temperature zone
KD91–Xinglong, Yi'an	4.88	45.83	17.12	36	Third accumulated temperature zone
KD91–Shengfeng Seed Industry, Wudalianchi	4.62	47.59	16.71	38.5	Fourth accumulated temperature zone
KD91–Hailun	4.98	47.14	17.38	38.1	Transition zone between the third and fourth accumulated temperature zones
KD91–Dalong Seed Industry, Bei'an	4.98	46.61	16.63	37.7	Fourth accumulated temperature zone
KD91–Shuanghe Farm, Gannan	4.66	46.2	17.94	37.6	Second accumulated temperature zone
KD91–Keshan 307 Field	4.98	43.7	18.17	34.3	Third accumulated temperature zone
KD91–Jiamusi Branch	5.19	44.94	18.68	36.2	Second accumulated temperature zone
KD91–Wudalianchi planting site	4.85	44.53	17.42	34.8	Fourth accumulated temperature zone
KD91–Yi'an County planting site	4.68	46.15	16.93	37.8	Third accumulated temperature zone

Note: The final column in the source table was labeled "longitude and latitude", but the entries describe accumulated temperature zones; the English heading therefore reflects the actual data content.
