# Supplementary material for: Contrasting Seed Protein Phenotypes Are Associated with Nodulation and Rhizosphere Microbiome Assembly in Soybean
Source: Microorganisms. 2026 Oct 5;14(10):2266. doi: 10.3390/microorganisms14102266 (PMC13707409; doi:10.3390/microorganisms14102266)
Supplement: Supplementary file 1 [file microorganisms-14-02266-s001.zip › Supplementary Table S3.pdf]

Table S3. Topological properties of rhizosphere bacterial and fungal co-occurrence networks in high- and low-protein soybean groups.

(A)Topological properties of the rhizosphere bacterial co-occurrence networks.

Network properties	H	L
Number of nodes	395	452
Number of edges	2492	2664
Proportion of positive edges	0.994	0.984
Proportion of negative edges	0.006	0.016
Edge density	0.032	0.026
Average degree	12.617	11.787
Modularity	0.829	0.848
Average path length	5.60	8.23

(B)Topological properties of the rhizosphere fungal co-occurrence networks.

Network properties	H	L
Number of nodes	573	751
Number of edges	10061	15526
Proportion of positive edges	0.999	0.999
Proportion of negative edges	0.001	0.001
Edge density	0.061	0.055
Average degree	35.117	41.348
Modularity	0.804	0.863
Average path length	6.89	4.79

Note: H and L represent the high-protein and low-protein soybean groups, respectively. Co-occurrence networks were constructed based on Spearman's rank correlations, and only significant associations with $|R| > 0.6$ and $P < 0.05$ were retained. Network properties include the number of nodes, number of edges, proportions of positive and negative edges, edge density, average degree, modularity, and average path length.
